# Supplementary material for: Identifying minimal risk factors for adolescent suicidal ideation and suicide attempts: A machine learning-optimized approach
Source: PLoS One. 2026 Apr 6;21(4):e0346050. doi: 10.1371/journal.pone.0346050 (PMC13052902; doi:10.1371/journal.pone.0346050)
Supplement: Attachment — Submitted filename: PONE-D-25-47059_Revision 1_Response to Reviewers.pdf [file pone.0346050.s002.pdf]

Reviewer 1's comments

The rationale for this study was the fact that "suicide is the 3rd leading cause of death for adolescents world-wide." It used data from the Korea Youth Risk Behavior Web-based Survey (2022–2023, Korea Disease Control and Prevention Agency, KDCA), including 90,217 adolescents aged 12–18 years. Suicidal: yes=11,838, no=78,975 (6.67%); attempts 2,191.

Data were analyzed via machine learning (Random Forest, RF) model, which they note is superior to meta-analysis. They used multidimensional risk factors (psychological, sociological, physical illness, demographic) spanning sociodemographic, physical and mental health, and behavioral domains. Sensitivity and specificity were assessed using the AUC method. "Sadness, loneliness, anxiety, and stress were identified as optimal features, achieving strong results in identifying suicidal ideation and predicting suicide attempts" == AUC 92%, Sens 94%, Spec 90%.

Cramer's V was used to define effect size of categoricals, Cohen's-d for effect size of continuous variables.

[how was suicidal vs. non-suicidal assessed?]

Of note, statistical risk factors by effect size were: Anxiety > Loneliness > Stress > Sleep > Sadness. These all had AUC >78%. While alcohol and smoking had very low effect size. 8 factors (sex, family economic status, sleep quality, self-reported health status, sadness, stress, anxiety, and loneliness) were statistically significant ($p < 0.05$) and relevant to clinical practice. Five statistical models comprised various combinations of these factors, with the most predictive (5) having sens=93.5%, spec= 90.3%

One wonders whether physical or sexual abuse was asked about in this large survey, and if so, what effect it would have on the results. Otherwise, the stated limitations are reasonable.

Fig. 3 displays factors that were internal to the machine learning method but are presented in a way that is unfamiliar to readers accustomed to reading AUC curves. I suggest that the authors either add a traditional AUC curve (Sensitivity vs. 1 – Specificity) for each of the 8 factors in their analysis, with all 8 AUCs overlaid on the same graph, or replace Fig. 3 with the latter AUC curve.

Overall, this paper is an excellent guide for those working with suicidal adolescents

Response:

We sincerely appreciate the reviewer's thorough evaluation and constructive feedback. Below, we have restated each comment (in bold) and provided detailed responses (in regular text). All corresponding revisions in the manuscript have been incorporated and are highlighted in grey.

Comments:

1. How was suicidal vs. non-suicidal assessed?

Response:

We thank the reviewer for pointing out the need for clearer clarification of the classification criteria.
In the revised manuscript, we have now explicitly described how suicidal ideation and suicide attempts were defined based on the KYRBS survey questions. Specifically, suicidal ideation and non-suicidal groups were classified according to responses to the following KYRBS items (e.g., During the past 12 months, have you ever seriously thought about committing suicide?; During the past 12 months, have you ever made a specific plan to commit suicide?; and During the past 12 months, have you ever attempted suicide?).

Participants who responded "Yes" to either suicidal thoughts or suicide planning were categorized into the suicidal ideation group, whereas those who answered "No" to both questions were classified as the non-suicidal group. For the analysis of suicide attempts, participants who responded "Yes" to the suicide attempt question were classified into the suicide attempt group, and those who answered "No" were classified as non-attempt. This clarification has been added to the Data Source, Participant

Selection, and Multidimensional Risk Factors section.

2. One wonders whether physical or sexual abuse was asked about in this large survey, and if so, what effect it would have on the results.

Response:

We examined the available variables from the KYRBS dataset and found no items specifically assessing physical or sexual abuse, such as sexual victimisation or non-partner violence. Because these trauma-related exposures are known risk factors for suicidal ideation and suicide attempts, the absence of such items in the dataset may limit the comprehensiveness of our risk-factor assessment. We have therefore added a statement in the Limitations section to that effect and recommend that future research incorporate datasets which include trauma or abuse-related items.

3. Fig. 3 displays factors that were internal to the machine learning method but are presented in a way that is unfamiliar to readers accustomed to reading AUC curves. I suggest that the authors either add a traditional AUC curve (Sensitivity vs. 1 – Specificity) for each of the 8 factors in their analysis, with all 8 AUCs overlaid on the same graph, or replace Fig. 3 with the latter AUC curve.

Response:

We greatly appreciate this valuable suggestion. Following the reviewer's recommendation, we have revised Figure 3 to include a traditional receiver operating characteristic (ROC) curve presentation. The updated figure now shows the overlaid ROC curves (Sensitivity vs. 1 – Specificity) for each model. We have also updated the corresponding Results subsection and Figure 3 legend to reflect these changes.
